# Folate receptor alpha expression associates with improved disease-free survival in triple negative breast cancer patients

**DOI:** 10.1038/s41523-020-0147-1

**Published:** 2020-02-04

**Authors:** Nadine Norton, Bahaaeldin Youssef, David W. Hillman, Aziza Nassar, Xochiquetzal J. Geiger, Brian M. Necela, Heshan Liu, Kathryn J. Ruddy, Mei-Yin C. Polley, James N. Ingle, Fergus J. Couch, Edith A. Perez, Minetta C. Liu, Jodi M. Carter, Roberto A. Leon-Ferre, Judy C. Boughey, Elizabeth B. Somers, Krishna R. Kalari, Daniel W. Visscher, Matthew P. Goetz, Keith L. Knutson

**Affiliations:** 1 Department of Cancer Biology, Mayo Clinic, Jacksonville, FL 32224 USA; 2 Department of Health Sciences Research, Mayo Clinic, Rochester, MN 55905 USA; 3 Department of Pathology, Mayo Clinic, Jacksonville, FL 32224 USA; 4 Department of Oncology, Mayo Clinic, Rochester, MN 55905 USA; 5 Department of Laboratory Medicine and Pathology, Mayo Clinic, Rochester, MN 55905 USA; 6 Division of Hematology and Oncology, Mayo Clinic, Jacksonville, FL 32224 USA; 7 Department of Surgery, Mayo Clinic, Rochester, MN 55905 USA; 8 Eisai, Inc, Exton, PA 19341 USA; 9 Molecular Pharmacology and Experimental Therapeutics, Mayo Clinic, Rochester, MN 55905 USA; 10 Department of Immunology, Mayo Clinic, Jacksonville, FL 32246 USA

**Keywords:** Breast cancer, Prognostic markers

## Abstract

Triple negative breast cancer (TNBC) comprises 15–20% of all invasive breast cancer and is associated with a poor prognosis. As therapy options are limited for this subtype, there is a significant need to identify new targeted approaches for TNBC patient management. The expression of the folate receptor alpha (FRα) is significantly increased in patients with TNBC and is therefore a potential biomarker and therapeutic target. We optimized and validated a FRα immunohistochemistry method, specific to TNBC, to measure FRα expression in a centrally confirmed cohort of 384 patients with TNBC in order to determine if expression of the protein is associated with invasive disease-free survival (IDFS) and overall survival (OS). The FRα IHC demonstrated exceptional performance characteristics with low intra- and interassay variability as well as minimal lot-to-lot variation. FRα expression, which varied widely from sample to sample, was detected in 274 (71%) of the TNBC lesions. In a multivariable model adjusted for baseline characteristics, FRα expression was associated with improved IDFS (HR = 0.63, *p* = 0.01) but not with OS. The results demonstrate the potential of targeting the FRα in the majority of TNBC patients and suggest that variable expression may point to a need to stratify on FRα expression in clinical studies.

## Introduction

Triple negative breast cancer (TNBC) occurs in ~15–20% of all patients with invasive breast cancer and is associated with a poor prognosis.^1^ Compared with other subtypes, patients with TNBC have an increased likelihood of distant recurrence, especially those with residual disease after neoadjuvant chemotherapy (NAC).^2^ As current treatment options are limited, there is a significant need to identify new targeted approaches for TNBC.

Folate receptor alpha (FRα) is a promising biomarker and therapeutic target for ovarian, breast, brain, lung, and colorectal cancers.^3–8^ Our previous work in large clinically relevant breast cancer cohorts demonstrated substantial FRα expression at the level of both mRNA (The Cancer Genome Atlas, *N* = 843) and protein (Mayo Clinic Tumor Registry, *N* = 131), with significantly increased expression in TNBC as compared with ER+ and HER2+ tumors.^9^ Prior small studies examining FRα expression in unselected cohorts have demonstrated an association between FRα expression and poor prognosis^10,11^ and relatively small studies have observed significant correlation of FRα expression and disease-free survival.^12^

At the biological level, folate is a necessary component of cell metabolism. Therefore, FRα overexpression may confer a growth advantage to tumors by increasing folate uptake and/or may affect cell proliferation via alternative cell signaling pathways.^13–15^ In vitro data from our own laboratory demonstrated that FRα expression influences the growth of triple negative cell lines, and its overexpression promoted folate uptake and provided a selective growth advantage in low folate conditions.^9^ In the same study, inhibition of FRα significantly reduced the cell growth of established TNBC cell lines and the magnitude of the effect was proportional to their original mRNA expression level. Therefore, patients with TNBC expressing high FRα may benefit from targeted anti-FRα therapy.

In the current study we developed and optimized an immunohistochemistry method to score FRα and used it in a centrally confirmed cohort of TNBC to test the association of FRα protein expression with invasive disease-free survival (IDFS) and overall survival (OS).

## Results

### The FRα-specific staining protocol demonstrated outstanding performance

The antibody was tested at 1:50, 1:100, 1:200 dilutions on an optimization TMA. No staining was observed (i.e., H-scores were all 0) for all liver and spleen tissue (Supplementary Fig. S1). At an antibody concentration of 1:100, of the normal breast tissue cores (*n* = 18), H-scores ranged from 0 to 220, mean H-score = 20 ± 56 (±SD). At an antibody concentration of 1:100, of the serous ovarian tissue cores used as positive controls (*n* = 18), the H-score ranged from 190 to 300, mean H-score 245 ± 57. At 1:100, the H-scores for the 19 TNBC specimens ranged from 0 to 280. At this dilution, 3 of 19 (16%) tumors showed zero staining (H-score of 0 for all three replicate punches). Sixteen of 19 (84%) of patients had a mean H-score > zero (Supplementary Fig. S2).

To characterize intra-assay variability, three adjacent 5-μm sections were taken from the blocks of 11 TNBC, three serous ovarian cancer, three liver and three spleen specimens to represent a range of intensities (based on observed H-scores from the optimization TMA). Three 5-μm sections were processed at antibody dilution of 1:100 in the same batch with the same reagents at the same time (Supplementary Fig. S3). Intra-assay variability across each of three 5-μm sections from the same specimen was 0, demonstrating high precision.

One 5-μm section, adjacent to those used in Supplementary Fig. S3, was taken from the blocks and stained on a separate day to those taken for intra-assay variability. Sections were processed at antibody dilution of 1:100 in the same batch with the same reagents, same technician and scored by the same pathologist. Linear regression analysis of H-scores on same samples (same core) processed at two different times showed a correlation of *R*^2^ = 0.86 (Supplementary Fig. S4). Thus, the protocol demonstrated excellent intra-assay variation.

We observed high reproducibility between two different antibody lots 13J4007 and 13J4008, when testing the same 1:100 dilution lot of 13J4007 (used for all 384 TNBC patients in this study) against a range of dilutions of a newer lot, 13J4008, with correlations ranging *R*^2^ = 0.80–0.95 (Supplementary Fig. S5).

To assess for FRα antigen stability, two adjacent 5-μm sections were taken from the blocks of TNBC patients: TN1, TN2, TN3, TN5, TN7, TN8, TN10, TN13, TN14, TN 17, and TN19, serous ovarian patients Sec1, Sec2, and Sec6, liver and spleen to represent a range of intensities (based on observed H-scores) at three different time points, day 0, day 15, and day 30. One section from each sample at each time point was stored at room temperature and a paired sample was stored at 4 °C. All sections were stained on day 30 with antibody from lot #13J40007 at a 1:100 dilution.

For sections stored at room temperature, we observed a good correlation of H-scores between samples stored for 15 and 30 days compared with those that were stained immediately after the slides was cut (*R*^2^ = 0.93 and 0.88, respectively) (Supplementary Fig. S6 panels A–C), highly similar to correlations observed for interassay and antibody lot experiments described above. We also observed good correlation for samples stored at 4 °C (*R*^2^ ranged 0.83–0.89) (Supplementary Fig. S6 panels D, E). It was concluded that FRα is stable for at least 30 days at ambient temperature and when refrigerated.

### Patient characteristics

Once optimized and validated, we evaluated FRα expression in our study cohort which consisted of 384 women with centrally confirmed TNBC derived from our internal TNBC patient registry and repository. The patient characteristics are shown in Table 1. The mean age of participants was 56 years, 50% of patients had tumor size ≤ 2.0 cm, and 64% were node-negative. The median follow-up for these patients was 12.7 years (interquartile range: 8.9–18.5 years).

**Table 1:** Patient and tumor characteristics (*N* = 384).

Parameter	Values
*Age (continuous)*	
Mean (SD)	55.6 (13.7)
Q1, median, Q3	45, 54.4, 65.8
Range	29.3–88.4
*Menopausal status*	
Post	227 (59%)
Pre/Peri	157 (41%)
*Tumor size*	
T1 (0.1–2.0 cm)	191 (49.7%)
T2 (2.1–5.0 cm)	169 (44%)
T3/4 (5.1+ cm)	23 (5.99%)
Unknown	1 (0.26%)
*Nodal status*	
N0	245 (64%)
N1	81 (21%)
N2	32 (8%)
N3	22 (6%)
NX	4 (1%)
*Histology*	
Invasive carcinoma NST	253 (66%)
Metaplastic carcinoma NST	30 (8%)
Carcinoma with apocrine differentiation	27 (7%)
Carcinoma with medullary features	74 (19%)
*Nottingham grade*	
1	2 (0.521%)
2	28 (7.29%)
3	354 (92.2%)
*Ki-67 grouping*	
≤15%	75 (19.5%)
15.1–30%	62 (16.1%)
>30%	246 (64.1%)
Unknown	1 (0.26%)
*Stromal TILs*	
0–10%	125 (33%)
10–20%	85 (22%)
20–40%	86 (22%)
>40%	85 (22%)
Unknown	3 (1%)
*FRα H-score (continuous)*	
Mean (SD)	77.1 (85.5)
Q1, median, Q3	0, 49, 133
Range	0–300
*FRα H-score group (zeros and rest by thirds)*	
0	110 (28.6%)
0.25–52.5	89 (23.2%)
52.5–140	93 (24.2%)
140–300	92 (24%)
*Type of breast surgery*	
Mastectomy	192 (50%)
Lumpectomy	192 (50%)
*Adjuvant chemotherapy*	
Yes	217 (56.5%)
No	118 (30.7%)
Unknown	49 (12.8%)
*Adjuvant radiotherapy*	
Yes	182 (47.4%)
No	146 (38%)
Unknown	56 (14.6%)

*NST* no special type, *FRα* folate receptor alpha.

### FRα is expressed in a high percentage of TNBCs

**Fig. 1:**
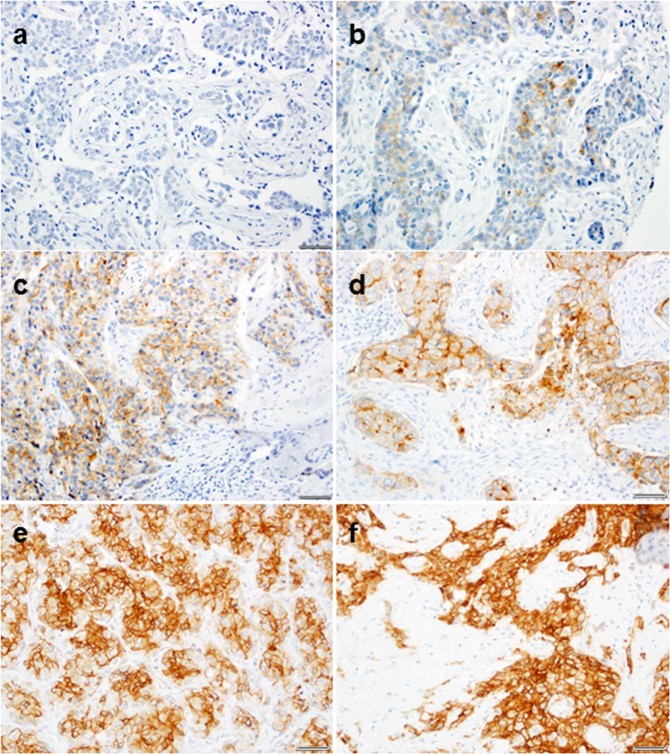
Representative FRα staining of triple negative breast tumors (20×). Membrane staining was scored as negative (0), weak (1+), moderate (2+), and strong (3+). The percent of cells within each tissue core stained at each intensity were recorded to calculate an H-score for each sample. The H-score for staining each sample was defined as: H-score = 0* (% at 0) + 1* (% at 1+) + 2* (% at 2+) + 3* (% at 3+). **a** H-score = 0: (3 × 0 + 2 × 0 + 3 × 0), **b** H-score = 50: (3 × 0 + 2 × 0 + 1 × 50), **c** H-score = 120: (3 × 0 + 2 × 20 + 1 × 80), **d** H-score = 140: (3 × 0 + 2 × 40 + 1 × 60), **e** H-score = 200: (3 × 20 + 2 × 60 + 1 × 20), **f** H-score = 300: (3 × 100 + 2 × 0 + 1 × 0). Scale bar is 50 µm.

We observed strong correlations between H-scores for the same core that was typed by the same pathologist 2 weeks apart with *R*^2^ values of 0.88, 0.94, 0.91, 0.87, and 0.85 for TMAs 1–5, respectively. For patients represented by multiple cores (*N* = 370), the correlation of H-scores was *R*^2^ = 0.68. The mean H-score was 77 with 110 of 384 (29%) participants having H-Score value of 0 (Table 1). Representative images are shown in Fig. 1. The mean stromal TIL count was 27.6%. There was no statistically significant association between H-score and stromal TIL count (*p* = 0.25).

### Increased FRα expression is associated with improved invasive disease-free survival (IDFS)

**Fig. 2:**
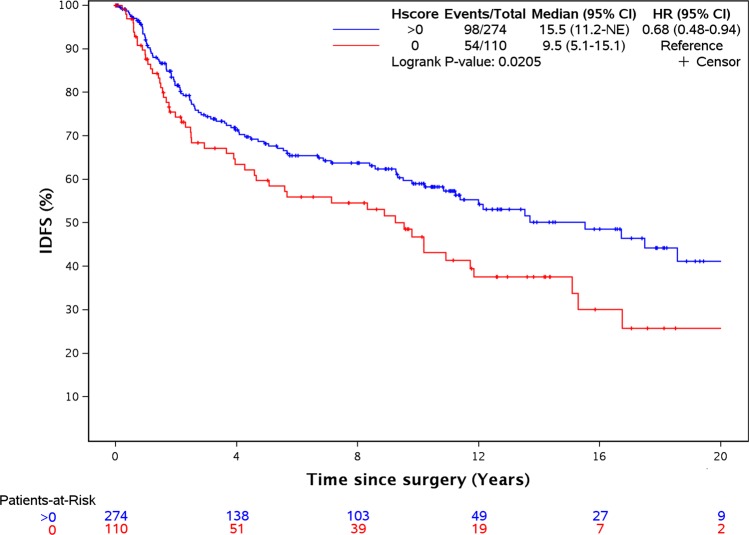
FRα expression correlates with IDFS. Kaplan–Meier analysis of invasive disease-free survival in patients with FRα expression >0 compared with patients with no expression.

In a univariable analysis (Table 2), stromal TILs (HR = 0.88, per 10% increase, *p* = 0.002), nodal status (overall *p*-value across all levels = 0.0006), and FRα H-scores (>0 vs 0; HR = 0.68; *p* = 0.02, Fig. 2) were associated with IDFS. The median IDFS (95% CI) for women with 0–10%, 10–20%, 20–40%, and >40% stromal TIL were 9.2 years (5.3–17.5), 10.9 years (3.8-NE (not evaluable)) 12.0 years (8.6-NE), and 20.4 years (11.8-NE), respectively.

**Table 2:** Univariable analysis of patient characteristics and FRα H-score with invasive disease-free survival and overall survival.

	IDFS		OS	
	HR (95% CI)	*P*-value	HR (95% CI)	*P*-value
*Age*				
<45.0	1.0 (ref)		1.0 (ref)	
45.0–54.3	0.85 (0.54–1.32)	0.46	0.83 (0.48–1.41)	0.483
54.4–65.7	0.92 (0.58–1.46)	0.72	1.52 (0.93–2.49)	0.091
65.8–88.4	1.44 (0.92–2.26)	0.11	3.01 (1.92–4.70)	<0.001
*Menopausal status*				
Pre/peri	1.0 (ref)		1.0 (ref)	
Post	1.31 (0.94–1.81)	0.11	2.37 (1.66–3.38)	<0.001
*Tumor size*				
T1 (0.1–2.0 cm)	1.0 (ref)		1.0 (ref)	
T2 (2.1–5.0 cm)	0.94 (0.68–1.32)	0.73	1.11 (0.80–1.55)	0.53
T3/4 (5.1+ cm)	1.7 (0.94–3.06)	0.08	1.95 (1.10–3.46)	0.02
*Nodal status*				
N0	1.0 (ref)		1.0 (ref)	
N1	1.36 (0.92–2.02)	0.13	1.59 (1.06–2.40)	0.03
N2	1.84 (1.1–3.06)	0.02	2.64 (1.63–4.28)	<0.0001
N3	3.44 (2–6.27)	<0.0001	3.59 (2.12–6.09)	<0.0001
NX	3.58 (0.88–14.60)	0.08	6.96 (2.53–19.20)	0.0002
*Histology*				
Invasive carcinoma NST	1.0 (ref)		1.0 (ref)	
Metaplastic carcinoma NST	0.96 (0.53–1.74)	0.88	0.63 (0.30–1.28)	0.20
Ca. with apocrine differentiation	1.15 (0.64–2.05)	0.64	1.11 (0.64–1.94)	0.71
Ca. with medullary features	0.60 (0.38–0.96)	0.03	0.60 (0.38–1.28)	0.03
*Nottingham grade*				
1	1.0 (ref)		1.0 (ref)	
2	0.49 (0.11–2.14)	0.34	0.568 (0.13–2.49)	0.45
3	0.32 (0.08–1.32)	0.12	0.398 (0.10–1.61)	0.2
*Ki-67 grouping*				
≤15%	1.0 (ref)		1.0 (ref)	
15.1–30%	0.76 (0.44–1.33)	0.34	0.56 (0.32–1.00)	0.05
>30%	1.06 (0.71–1.60)	0.77	0.84 (0.57–1.24)	0.38
*Stromal TILs (per 10% increment)*	0.88 (0.81–0.95)	0.002	0.88 (0.81–0.95)	0.001
*FRα H-score*				
0	1.0 (ref)		1.0 (ref)	
0.25–52.5	0.55 (0.35–0.88)	0.01	0.59 (0.37–0.93)	0.02
52.5–140	0.64 (0.41–1.00)	0.05	0.79 (0.51–1.21)	0.28
140–300	0.84 (0.56–1.26)	0.4	0.901 (0.60–1.38)	0.65
*FRα H-score*				
≤49.375 (median)	1.0 (ref)		1.0 (ref)	
>49.375 (median)	0.94 (0.69–1.30)	0.73	1.07 (0.78–1.47)	0.67
*FRα H-score*				
0	1.0 (ref)		1.0 (ref)	
>0	0.68 (0.49–0.94)	0.02	0.75 (0.54–1.06)	0.10
*Surgery type*				
Lumpectomy	1.0 (ref)		1.0 (ref)	
Mastectomy	1.06 (0.77–1.46)	0.72	1.35 (0.97–1.88)	0.07
*Adjuvant chemotherapy*				
No	1.0 (ref)		1.0 (ref)	
Yes	0.79 (0.56–1.10)	0.16	0.7 (0.49–0.99)	0.04
Unknown	1.18 (0.51–2.74)	0.71	1.57 (0.96–2.59)	0.07
*Adjuvant radiotherapy*				
No	1.0 (ref)		1.0 (ref)	
Yes	0.94 (0.67–1.30)	0.7	0.73 (0.52–1.04)	0.08
Unknown	1.06 (0.42–2.64)	0.91	1.5 (0.92–2.45)	0.1

*NST* no special type, *FRα* folate receptor alpha.

In separate analyses of FRα H-scores, we compared the outcome of patients in each of the positive H-score tertiles (0.25–52.5, 52.5–140, and 140–300) against patients with zero expression (H-score 0) (Table 2). In patients with relatively low levels of FRα expression (H-scores 0.25–52.5 and 52.5–140), IDFS was significantly improved compared with patients with H-score of 0 (HR 0.55, *p* = 0.01 and HR 0.64, *p* = 0.05, respectively). The group of patients with the highest FRα expression (H-scores 140–300) did not show significantly improved survival relative to patients with no FRα expression, although the HR was in the same direction as the low FRα groups (HR 0.84, *p* = 0.4).

In the multivariable analysis (Table 3), FRα H-score (>0 vs 0, *p* = 0.01), adjuvant chemotherapy (vs no chemotherapy, *p* = 0.03), stromal TILs (per 10% increment, *p* = 0.003), and nodal status (*p* < 0.0001) were significantly associated with IDFS.

**Table 3:** Multivariable analysis of FRα H-score with invasive disease-free and overall survival.

	IDFS		OS	
	HR (95% CI)	*P*-value	HR (95% CI)	*P*-value
*Age*				
<45	1.0 (ref)		1.0 (ref)	
45–54.3	0.65 (0.39–1.08)	0.09	0.62 (0.33–1.17)	0.14
54.4–65.7	0.55 (0.27–1.10)	0.09	0.80 (0.36–1.77)	0.58
65.8–88.4	0.73 (0.35–1.53)	0.41	1.29 (0.58–2.88)	0.53
*Menopausal status*				
Pre/Peri	1.0 (ref)		1.0 (ref)	
Post	1.66 (0.95–2.91)	0.08	2.00 (1.02–3.91)	0.04
*Tumor size*				
T1 (0.1–2.0 cm)	1.0 (ref)		1.0 (ref)	
T2 (2.1–5.0 cm)	1.17 (0.81–1.71)	0.4	1.35 (0.92–1.96)	0.12
T3/4 (5.1+ cm)	1.84 (0.92–3.69)	0.08	1.90 (0.97–3.74)	0.06
*Nodal status*				
N0	1.0 (ref)	0.008	1.0 (ref)	
N1	1.90 (1.18–3.04)	0.008	2.72 (1.67–4.43)	<0.001
N2	2.33 (1.27–4.29)	0.007	3.54 (1.96–6.37)	<0.001
N3	6.02 (3.05–11.90)	<0.001	8.95 (4.65–17.24)	<0.001
NX	2.44 (0.53–11.36)	0.25	3.77 (1.21–11.74)	0.02
*Histology*				
Invasive carcinoma NST	1.0 (ref)		1.0 (ref)	
Metaplastic carcinoma NST	0.82 (0.43–1.58)	0.55	0.50 (0.23–1.09)	0.08
Ca. with apocrine differentiation	0.53 (0.22–1.28)	0.16	0.38 (0.16–0.91)	0.03
Ca. with medullary features	0.83 (0.49–1.41)	0.49	0.80 (0.47–1.37)	0.42
*Nottingham grade*				
1	1.0 (ref)		1.0 (ref)	
2	1.10 (0.23–5.27)	0.9	1.21 (0.25–5.91)	0.81
3	0.54 (0.11–2.55)	0.44	0.83 (0.18–3.84)	0.81
*Ki-67 grouping*				
≤15%	1.0 (ref)		1.0 (ref)	
15.1–30%	0.94 (0.51–1.72)	0.83	0.78 (0.42–1.45)	0.43
>30%	1.28 (0.77–2.11)	0.34	0.93 (0.58–1.49)	0.75
*Stromal TILs (per 10% increment)*	0.86 (0.78–0.95)	0.003	0.86 (0.78–0.95)	0.004
*FRα H-score*				
0	1.0 (ref)		1.0 (ref)	
>0	0.63 (0.44–0.91)	0.01	0.87 (0.60–1.26)	0.46
*Adjuvant chemotherapy*				
No	1.0 (ref)		1.0 (ref)	
Yes	0.61 (0.39–0.95)	0.03	0.44 (0.28–0.71)	<0.001
Unknown	1.27 (0.34–4.73)	0.73	1.07 (0.34–3.33)	0.91
*Adjuvant radiotherapy*				
No	1.0 (ref)		1.0 (ref)	
Yes	0.87 (0.51–1.48)	0.62	0.85 (0.50–1.43)	0.53
Unknown	1.12 (0.28–4.56)	0.87	0.87 (0.28–2.68)	0.81

*NST* no special type, *FRα* folate receptor alpha.

### FRα expression and overall survival (OS)

Univariable analyses of OS are shown in Table 2. Age (>65.8 compared with age < 45, *p* < 0.001), nodal status (any nodal positivity compared with N0, *p* < 0.001), adjuvant chemotherapy (compared with no chemotherapy, *p* = 0.04), tumor size > 5.0 cm (compared with 0.1–2.0 cm, *p* = 0.02), menopausal status (*p* < 0.001), and stromal TILs (*p* = 0.001) were associated with OS; with older age, post-menopausal, node-positive, low stromal TILs, and absence of adjuvant chemotherapy all being associated with decreased OS.

**Fig. 3:**
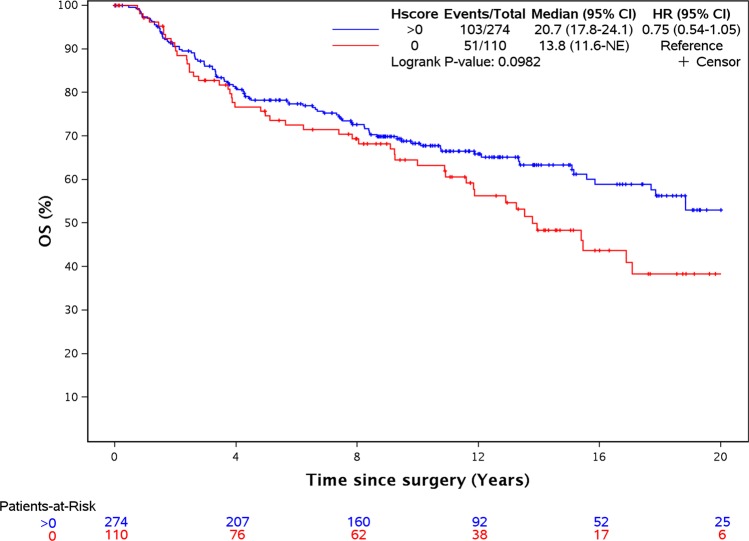
FRα expression does not correlate with OS. Kaplan–Meier analysis of overall survival survival in patients with FRa expression >0 compared with patients with no expression.

FRα H-score was not associated with OS (overall *p*-value = 0.11 comparing the four groups of H-scores). The patient group with H-score values 0.25–52.5 appeared to have longer survival times when compared with participants with values of 0. However, this association was not seen in patients with higher H-scores. Comparing H-scores of >0 vs 0 was also not statistically significant (HR = 0.75, *p* = 0.10) but showed a similar trend to that observed with IDFS (Fig. 3).

In the multivariable analysis (Table 3), FRα H-scores of >0 vs 0 were not statistically associated with OS (HR = 0.87, *p* = 0.46). Age (across all levels compared with age < 45, *p* < 0.04), nodal status (across all levels compared with N0, *p* < 0.001), adjuvant chemotherapy (all levels compared with no chemotherapy, *p* = 0.002), menopausal status (*p* = 0.04), and stromal TILs (*p* = 0.004) were associated with OS.

### Prognostic effect of FRα expression in chemotherapy treated and untreated patients

The TNBC cohort in this study contains a substantial number of patients (31%) that were not treated with adjuvant chemotherapy, allowing us to test if FRα expression was prognostic (yes/no) in patients treated with chemotherapy and separately in patients who were not treated with chemotherapy. In univariable analyses, FRα H-scores of >0 vs 0 were statistically associated with IDFS in patients that did not receive chemotherapy, HR = 0.54, *p* = 0.03, and this remained significant in multivariable analysis, HR = 0.50, *p* = 0.03. Univariable analysis of FRα H-scores of >0 vs 0 in patients that were treated with chemotherapy trended in the same direction, HR = 0.74, *p* = 0.18, and were significantly associated with IDFS in multivariable analysis, HR = 0.61, *p* = 0.04. The same analyses with OS were not statistically significant in patients treated or untreated with chemotherapy.

## Discussion

Using a rigorously tested immunostaining protocol in a large, well-annotated cohort of 384 centrally reviewed TNBC patients, we evaluated the association of FRα protein expression with IDFS and OS. To capture both the proportion of positively stained cells and the intensity of staining we used a quantitative H-score (range 0–300) approach and observed that the expression of FRα (H-score > 0) was associated with longer IDFS after controlling for other known prognostic factors in a multivariable analyses.

Our data are conflicting with the prior published studies of FRα expression in patients with breast cancer. To date, three published studies evaluated the association of FRα expression with outcome in unselected breast cancer patients^10–12^ with sample sizes ranging *N* = 63–429. Two of the studies that found an association between FRα and outcomes included mostly patients with ER+ tumors, and found no association with outcomes in the TNBC group. A third study found an association between increased FRα expression and worse DFS in 76 TNBC patients (HR, 2.61, *p* = 0.0497); however, in that study 80.3% of the TNBC cases evaluated were negative for FRα (defined as ≥5% positive staining).^12^ Although each of these studies used different criteria for scoring FRα positivity, between 20 and 30% of the patient samples were scored as FRα expressing and these patients demonstrated worse survival. This is in contrast with our study in which we observed better survival in ~71% of patients who expressed FRα at some level and we did not observe a worse outcome in the 30% of patients with the highest levels of FRα.

To put into context these conflicting findings, we draw a parallel to studies of FRα expression and survival in ovarian carcinoma which also demonstrate the importance of sample size, histological subtype, central review of IHC and further evidence that FRα positivity is associated with improved survival. A relatively small study of ovarian carcinoma (*N* = 91) showed association of increased FRα gene expression with worse survival,^16^ two IHC studies of mixed histological subtypes (*N* = 186 and *N* = 361, respectively) showed no association of FRα expression with survival^17,18^ and a subsequent larger study from the Ovarian Tumor Tissue Analysis consortium, demonstrated association of FRα positivity with improved survival, specifically in patients with high grade serous ovarian carcinoma (*N* = 1422).^19^ The Kobel study^19^ which showed improved survival in patients who had FRα expression was the largest study of FRα expression in ovarian cancer, the IHC was centrally reviewed and they used a similar scoring of FRα expression to our own, in that patients with absent or weak (<1%) staining were defined as negative and all other patients were recorded as positive. Similar criteria of FRα positivity were also used in a study of non-small cell lung cancer, in which H-scores ≥ 20 were associated with prolonged PFS (5.5 vs. 3.4 months; HR = 0.61; *P* = 0.0254) and improved OS (12.1 vs. 6.4 months; HR = 0.57; *P* = 0.0076).^20^ Finally, FRα positive expression is associated with better prognosis in a second study of non-small cell lung cancer^20,21^ and in pancreatic ductal adenocarcinoma.^22^

There are two possible mechanistic links between expression of FRα and better prognosis. First, one could hypothesize that tumors expressing FRα are more sensitive to chemotherapy. Huang showed that SKOV3 ovarian cancer cells overexpressing FRα were significantly more sensitive to cisplatin than controls.^23^ In addition, one could hypothesize that chemotherapy releases FRα antigens driving an immune response. This is consistent with our prior data where we prospectively tested for immunity in both breast and ovarian cancer patients using a panel of FRα-derived peptides representing potential T-cell epitopes.^24^ In that study, more than 70% of patients demonstrated immunity to at least one FRα peptide.

The limitations of our study are the retrospective nature and that patients were treated heterogeneously (56% of patients were treated with chemotherapy and 31% did not receive chemotherapy). However, this heterogeneity did allow us to determine the prognostic role of FRα in patients treated with surgery alone (no adjuvant chemotherapy) and separately in patients who were treated with chemotherapy, in which we observed that FRα H-scores of >0 were associated with better IDFS in both of these groups. Thus, although our study did not identify why FRα expression is associated with better outcome, our observation that FRα is also prognostic in untreated TNBC, generates a new hypothesis, that endogenous immune responses against FRα may also drive prognosis in TNBC. However, evaluation of a larger randomized cohort would be needed to test this hypothesis and if there is any interaction between chemotherapy and FRα expression in determining outcome, for which our optimized assay of FRα expression would be a useful tool.

What may be the most important finding in this study is that while >70% of TNBC patients showed at least some positivity for FRα, patients with FRα positive TNBC still exhibited disease recurrence, albeit at a lower rate compared with FRα negative patients, suggesting a substantial need to improve the therapeutic outcomes for this group. Regarding treatment strategies for this subset of patients, there are now several therapies directed at FRα which include monoclonal antibodies alone such as farletuzumab or as drug conjugates such as MOv18-IgG1 (and anti-FRα antibody conjugated with a Src inhibitor^25^), FRα engineered chimeric antigen receptor (CAR) T cells^26^ and a vaccine-based approach.^24^ In the present cohort, IDFS in patients who were positive for FRα was still declining after 10 years such that approaches that generate a durable response may be more appropriate. CAR-modified T cells, for example, have the capacity to persist as memory cells in vivo^27,28^ although recent data demonstrated that FRα CAR T cells mediated antitumor activity against established TNBC tumor when FRα is expressed at higher levels,^26^ which has significant implications for pre-selection of TNBC patients based on accurately defined FRα expression. Recent phase I clinical data from our group demonstrated that a FRα peptide vaccine elicited a durable (at least 12 months) T-cell response to the FRα peptides in 90% of patients, including both breast and ovarian cancer.^24^ Therefore, further augmenting immune responses to patients with TNBC may be of substantial therapeutic relevance, and a randomized phase II trial (including correlative studies of FRα expression with high performance IHC assay) is ongoing to test a FRα vaccine in patients with high risk, resected TNBC (NCT03012100).

## Methods

### Ethics approval and consent to participate

All breast cancer specimens were collected according to a protocol that was approved by the Mayo Clinic Institutional Review Board (IRB). The study was conducted in accordance with the U.S. Common rule with written, informed consent being obtained from each participating patient.

### Tissue microarrays (TMAs)

A tissue microarray (TMA) was constructed for the purpose of antibody optimization consisting of 1 mm core punches from deidentified waste tissue: 8 from liver and 8 from spleen, intended as negative controls; 3 replicate punches of each of 19 TNBC breast tumors; 3 replicate punches of each of 6 normal breast tissues and 3 replicate punches of each of 6 serous ovarian cancer, tissues intended as positive controls. Samples from this TMA and 5-μm sections from the same patients were used to optimize and test antibody dilution, intra- and interassay variability, reproducibility between different antibody lots and antigen stability.

Five TMAs with 1.0 mm cores, derived from TNBC surgical specimens, were constructed by the Mayo Clinic Pathology Research Core. Two cores from each tumor specimen were included per array and each array included controls of liver, normal breast, tonsil, cervix, and placenta. Four TMAs included 88 patient specimens and one TMA included 57 specimens. The arrays were created using the semi-automated Alphelys (Plaisir, France) Minicore tissue arrayer.

### Immunohistochemistry

Immunohistochemistry (IHC) was performed on FFPE tissue microarrays using a MACH4 Universal HRP-Polymer Detection Kit (Biocare Medical, Pancheco, CA) as previously described.^29^ FFPE TMA specimens were sectioned at 5 μm, placed on positively-charged glass slides and heated at 60 °C for at least 1 h. Slides were deparaffinized in sequential baths of xylene, transferred to sequential baths of 100% ethanol, followed by sequential baths of 95% ethanol and then rinsed in deionized (DI) water. The IHC procedure involves pretreatment of slides in Diva heat-induced epitope retrieval solution (Biocare Medical) inside a pressurized decloaking chamber with DI water and a pressurized incubation period at elevated temperature (125 °C at 16 psi for 30 s) followed by a 15 min of cooling to 95 °C. The slides were then cooled at room temperature, washed in sequential baths of Tris buffered Saline/0.1% Tween-20 wash buffer (TBST). Slides were blocked using Peroxidase-1 blocking solution (Biocare Medical), washed with TBST buffer and blocked with a serum-free universal blocking reagent. Slides were incubated with Anti-FRα murine monoclonal antibody, Clone 26B3.F2, Lot number 13J40007 (Eisai) at 1:100 dilution in antibody diluent (Dako, Carpinteria, CA) or with Bond Negative Mouse ready-to-use negative control antibody (Dako, for negative isotype tissue) for 60 min at room temperature. Slides were washed with TBST buffer and then incubated with MACH4 Mouse Probe Primary Antibody Enhancer (Biocare Medical) for 15 min, and then Universal Polymer-HRP reagent (Biocare Medical) for 20 min. After additional TBST washes, slides were incubated with a 3,3′-diaminobenzidine tetrahydrochloride (DAB) solution (Dako), rinsed and counter-stained with hematoxylin. Slides were washed with water, dehydrated with sequential baths each of 95 and 100% ethanol and then sequential baths of xylene before coverslips were applied.

### IHC scoring

Digital images of the stained TMA slides were obtained using an Aperio ScanScope Image Scanner (Aperio Technologies, Vista, CA). TMAs were evaluated using a semi-quantitative scoring method (Fig. 1). A pathologist (BY) scored membrane staining as negative (0), weak (1+), moderate (2+), and strong (3+) membrane staining. The percent of cells within each tissue core stained at each intensity were recorded to calculate an H-score for each sample. The H-score is a weighted score that captures both the proportion of positively stained cells and the intensity of staining, and thus is more representative of staining of the entire tumor section. The H-score for staining each sample was defined as: H-score = 0* (% at 0) + 1* (% at 1+) + 2* (% at 2+) + 3* (% at 3+). H-scores for duplicate patient cores were averaged. Duplicate patient cores were available for 370 patients with only 14 patients having a single core. For the 384 TNBC patients, each TMA was scored once by the study pathologist (BY) and then scored again three weeks later by the same pathologist. Analyses were based on the average of the two H-scores. The pathologist scoring the TMA was blinded to patient demographic and clinical outcome data.

### Study population and pathologic assessment

This cohort included 384 TNBC patients seen at the Mayo Clinic. Patients were identified through the Mayo Clinic, Rochester, MN database who underwent surgery for stage I–III BC between January 1, 1985 and December 31, 2012, and who were clinically HER2 negative or unknown and did not receive anti-HER2 therapy. Tissue sections from formalin-fixed, paraffin-embedded (FFPE) tumor blocks were obtained and centrally evaluated for ER, PR, and HER2 at the Mayo Clinic in the Pathology Research Core. ER and PR immunoreactivity were assessed using antibodies derived from clones 1D5 and PgR363, respectively (Dako), with staining ≥1% considered as positive. HER2 immunoreactivity was assessed using the HercepTest Kit (Dako, Carpinteria, CA) followed by fluorescence in situ hybridization (FISH, for IHC 2+ cases only), and categorized according to current ASCO/CAP guidelines.^30^ Stromal tumor-infiltrating lymphocyte (TIL) data were derived from our previously reported study that examined TILs according to the TILs Working Group recommendations.^31,32^

### Statistical methods

Correlations of FRα IHC parameters were tested by linear regression, *R*^2^. IDFS and OS were defined as per the STEEP classification.^33^ Patients who were event-free at the last date of disease evaluation were censored for IDFS. If death occurred >365 days after the last disease evaluation where the patient was found to be recurrence-free, IDFS was censored on the last date of disease evaluation.

The Kaplan–Meier method was used to estimate the distributions of IDFS and OS. The log-rank test was used to compare survival distributions among groups. Univariable Cox proportional hazards models were fit to assess the univariable association between each baseline variable and clinical outcomes. Multivariable Cox proportional hazards models were fit to assess the association between each baseline covariate with clinical outcomes while adjusting for the effects of other patient and disease characteristics. Results are expressed in hazard ratios (HRs) and 95% confidence intervals (CI).

Due to the skewness, the H-scores were split into four subgroups with the H-scores of 0 compared with the other three equally sized groups ([0.25–52.5), [52.5–140), and [140–300]) for the IDFS and OS analysis. H-scores were also analyzed by whether H-score existed or not (0 vs > 0) and by the median. Association between H-score and stromal TILs groups were assessed using general association and trend Chi-squared tests.

All statistical tests were two-sided. No statistical adjustement of multiple comparisons was made. In all cases, *p* values < 0.05 were considered statistically significant. The statistical analyses were carried out using SAS version 9.4 (SAS, Cary, NC).

### Reporting summary

Further information on research design is available in the Nature Research Reporting Summary linked to this article.

## Supplementary information

Supplementary Data

reporting summary

## Data Availability

The data generated and analyzed during this study are described in the following data record: 10.6084/m9.figshare.11549283.^34^ The .tif image files that make up Fig. 1, are part of the published article. Data supporting Figs 2 and 3 and Tables 1–3 are not publicly available in order to protect patient privacy, but can be made available on reasonable request from the corresponding author, as described in the metadata record above. The data supporting supplementary Figs 1–6 are available as part of the supplementary information.
