# Supplementary material for: Folate receptor alpha expression associates with improved disease-free survival in triple negative breast cancer patients
Source: NPJ Breast Cancer. 2020 Feb 4;6:4. doi: 10.1038/s41523-020-0147-1 (PMC7000381; doi:10.1038/s41523-020-0147-1)
Supplement: Supplementary file 1 — Supplementary Data [file 41523_2020_147_MOESM1_ESM.pdf]

Supplementary Information

Folate Receptor Alpha Expression is Associated with Improved Disease Free Survival in Triple Negative Breast Cancer

Nadine Norton[1], Bahaaeldin Youssef[1], David W. Hillman[2], Aziza Nassar[3], Xochiquetzal Geiger[3], Brian M. Necela[1], Heshan Liu[2], Kathryn J. Ruddy[4], Mei-Yin C. Polley[2], James N. Ingle[4], Fergus J. Couch[2,5], Edith A. Perez[6], Minetta C. Liu[4,5], Jodi M. Carter[5], Roberto A. Leon-Ferre[4], Judy C. Boughey[7], Elizabeth B. Somers[8], Krishna R. Kalari[2], Daniel W. Visscher[5], **Matthew P. Goetz[4,9], **Keith L. Knutson[10]

[1]Department of Cancer Biology, Mayo Clinic Jacksonville, FL 32224, [2]Department of Health Sciences Research, Mayo Clinic, Rochester, MN 55905, [3]Department of Pathology, Mayo Clinic Jacksonville, FL 32224, [4]Department of Oncology, Mayo Clinic, Rochester, MN 55905, [5]Department of Laboratory Medicine and Pathology, Mayo Clinic, Rochester, MN 55905, [6]Division of Hematology and Oncology, Mayo Clinic, Jacksonville, FL 32224, [7]Department of Surgery, Mayo Clinic, Rochester, MN 55905, [8]Eisai, Inc., Exton, PA 19341, [9]Molecular Pharmacology and Experimental Therapeutics, Mayo Clinic, Rochester, MN 55905, [10]Department of Immunology, Mayo Clinic, Jacksonville, FL 32246

**indicates co-senior authors.

Corresponding Author: Dr. Nadine Norton, Department of Cancer Biology, Griffin Building 212, Mayo Clinic, Jacksonville, FL 32224, Tel: 904-953-6352, email: norton.nadine@mayo.edu

SUPPLEMENTARY FIGURES AND LEGENDS

Supplementary Figure 1

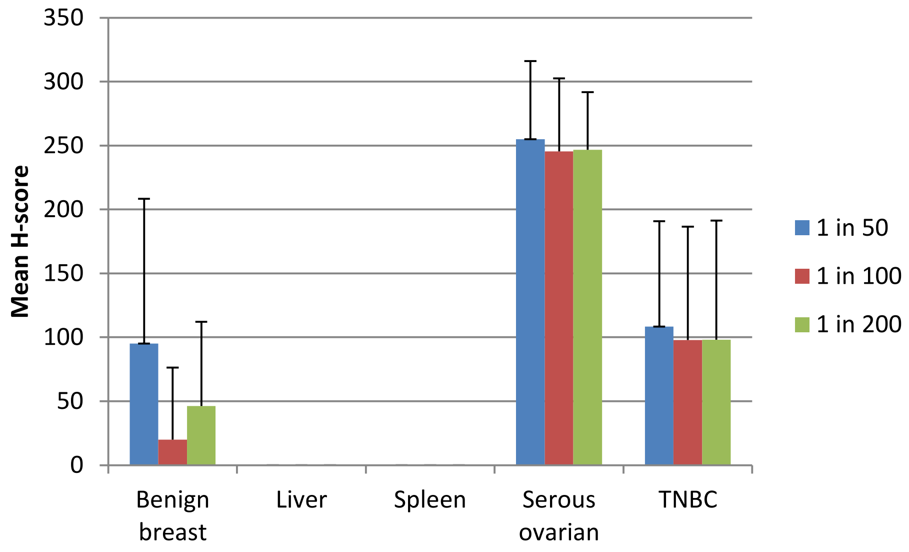

Fig. S1: The antibody (lot # 13J40007) was tested at 1:50, 1:100, 1:200 dilutions on an optimization TMA. H-score distributions were similar for each concentration. Error bars are standard deviation.

Supplementary Figure 2

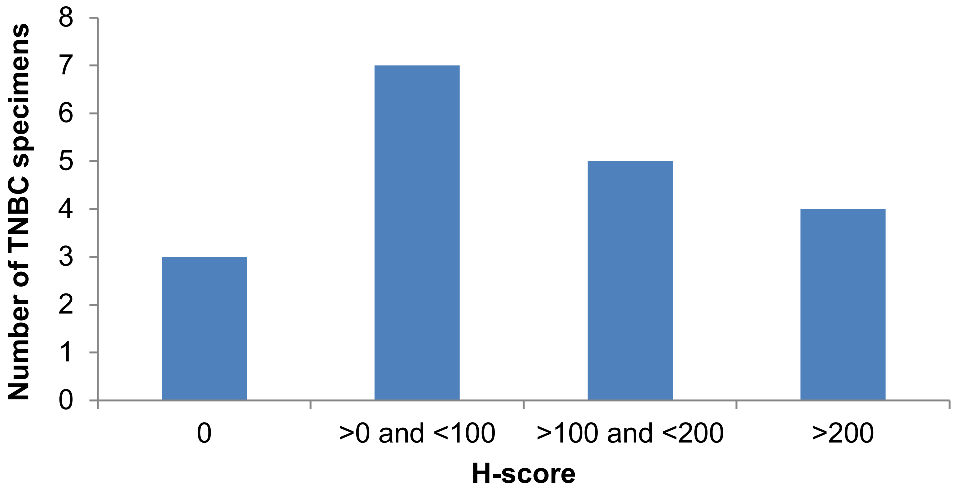

Fig. S2: FRα expression is heterogeneous in TNBC. Distribution of H-scores in 19 TNBC specimens at an antibody dilution of 1:100 using antibody lot # 13J40007. At 1:100, H-scores for 19 TN tumors ranged from 0 to 300. 3 /19 (16%) tumors showed zero staining (H-score of 0 for all three replicate punches) and 16 of 19 (84%) had a mean H-score >zero.

Supplementary Figure 3

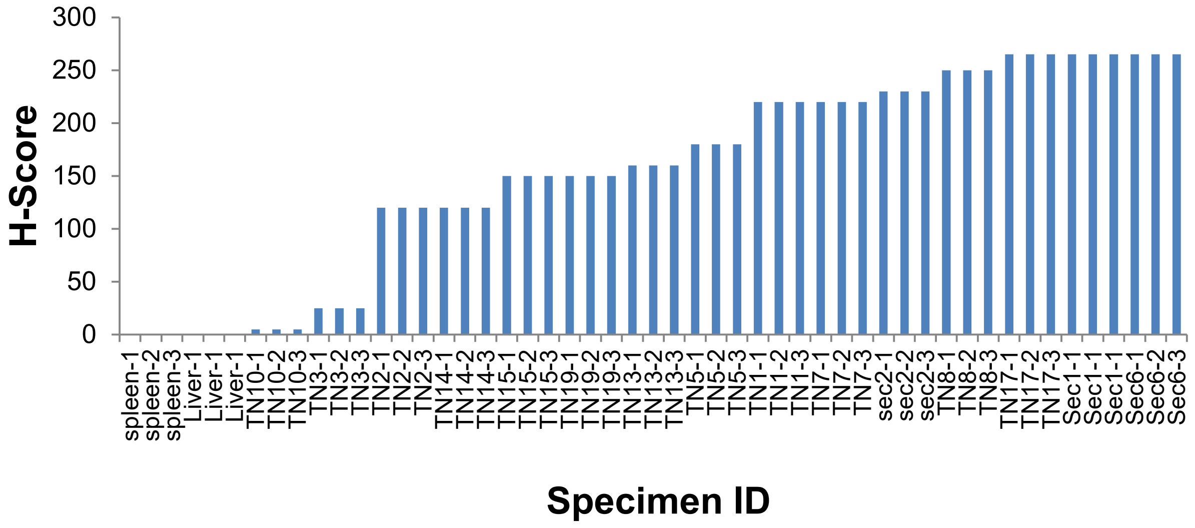

Fig. S3. Intra-assay variation in FRα IHC staining of TNBC sections is negligible. Shown are FRα H-scores of three 5 μm sections from TNBC specimens, serous ovarian cancer (Sec) and liver and spleen specimens, stained using antibody lot # 13J40007. Adjacent 5 μm were sections were taken from the blocks of TNBC specimens: TN1, TN2, TN3, TN5, TN7, TN8, TN10, TN13, TN14, TN 17 and TN19, serous ovarian (Sec) patients, Sec1, Sec2 and Sec6 and liver and spleen to represent a range of intensities.

Supplementary Figure 4

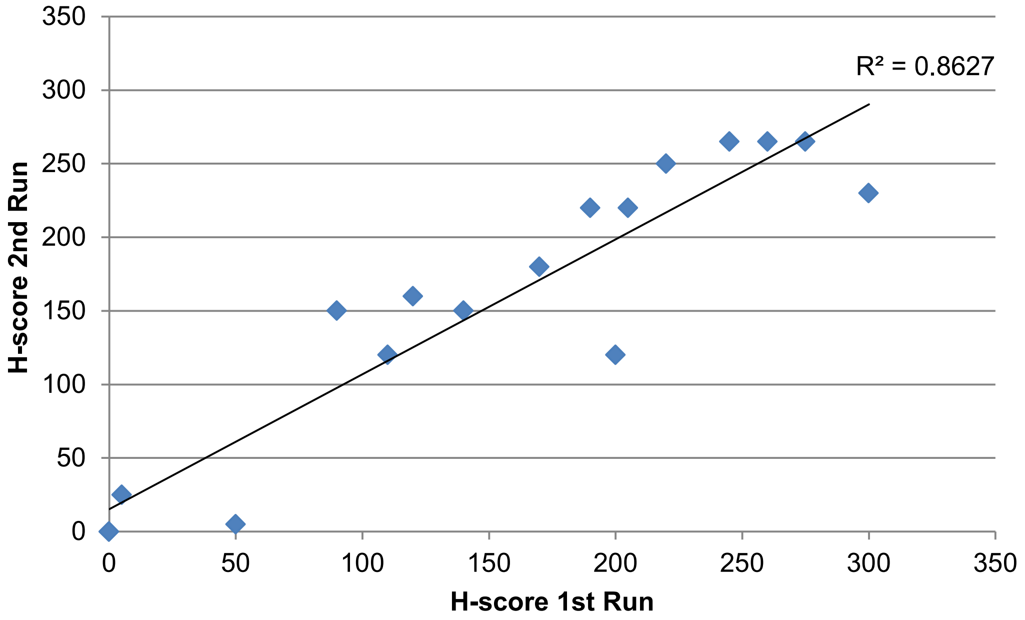

Fig. S4: Inter-assay variation in FRα IHC staining of TNBC sections is negligible Shown are coordinated FRα H-scores of two 5 μm sections from TNBC specimens, serous ovarian cancer (Sec) as well as liver and spleen specimens, stained using antibody lot # 13J40007. Adjacent 5 μm section were taken from the blocks of 11 TNBC specimens, three serous ovarian tumors, as well as liver and spleen to represent a range of intensities and stained on a separate day as those in Fig. S2. Sections were processed at antibody dilution of 1:100 in the same batch with the same reagents, same technician and scored by the same pathologist. Linear regression to assess correlation of H-scores on same samples processed at two different times showed a correlation of R^2=0.86.

Supplementary Figure 5

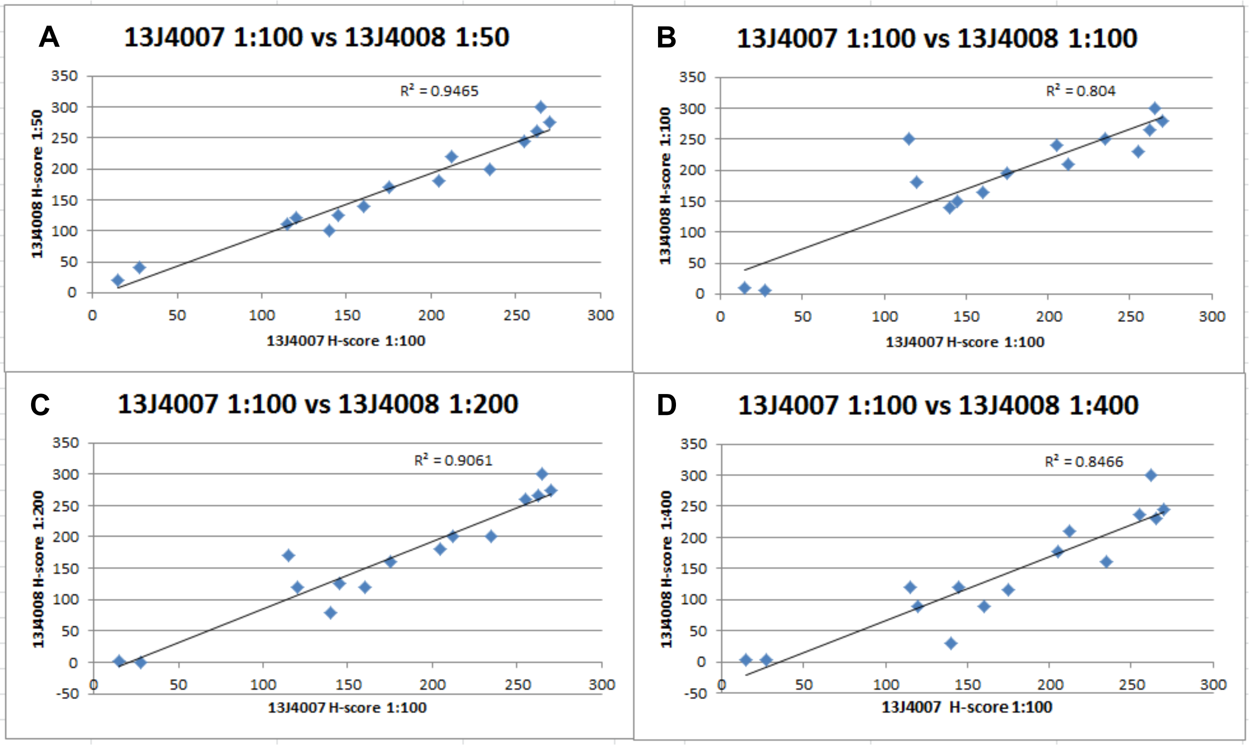

Fig. S5. FRα antibody lots showed high reproducibility. Adjacent 5 μm were section was taken from the blocks of TN patients: TN1, TN2, TN3, TN5, TN7, TN8, TN10, TN13, TN14, TN 17 and TN19, Serous Ovarian patients Sec 1, Sec2 and Sec6 and liver and spleen to represent a range of intensities (based on observed H-scores from the TMA in section 1). Sections were processed with a new lot of antibody (13J4008) at dilutions of 1:50 (A), 1:100 (B), 1:200 (C) and 1:400 (D) in the same batch with the same reagents, same technician and scored by the same pathologist. Linear regression was performed to assess correlation of H-scores on each dilution of the new antibody lot (13J4008) and compared against the optimized 1:100 dilution of the original antibody lot (13J4007). We observed correlations of R2 = 0.95, 0.80, 0.91 and 0.85 respectively.

Supplementary Figure 6

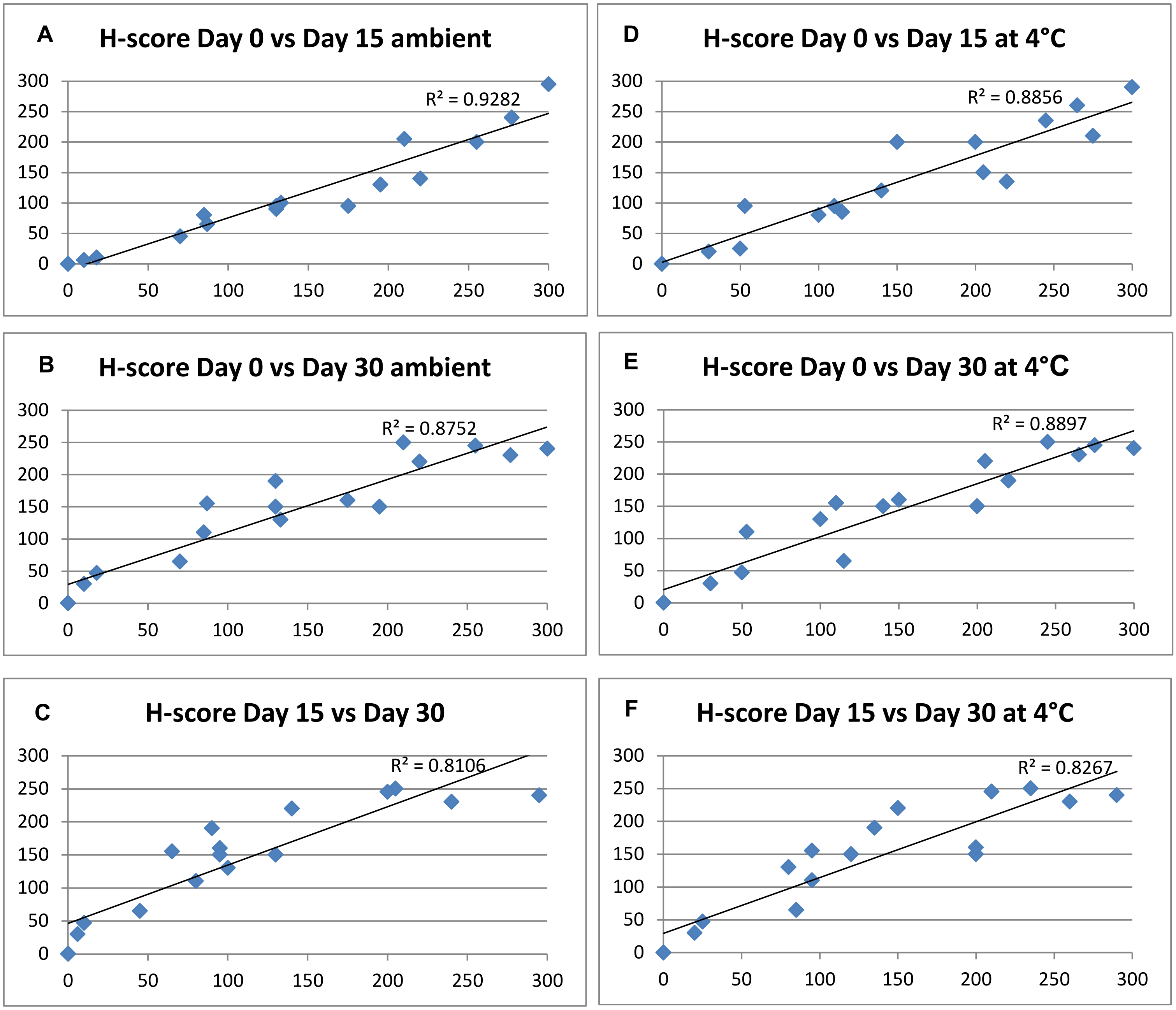

Fig. S6. FRα antibody was stable up to 30 days at room temperature and 4°C. For sections stored at room temperature and 4°C, we observed good correlation of H-scores between samples stored for 15 and 30 days compared to those that were stained immediately after the slides was cut. Correlation of H-score between samples stored for 30 days versus those immediately stained was highly similar to correlation observed for inter-assay and different antibody lot experiments described above.
